# Elucidating the Functional Roles of Helper and Cytotoxic T Cells in the Cell-Mediated Immune Responses of Olive Flounder (*Paralichthys olivaceus*)

**DOI:** 10.3390/ijms22020847

**Published:** 2021-01-15

**Authors:** Jae Wook Jung, Ae Rin Lee, Jaesung Kim, Young Rim Kim, Jassy Mary S. Lazarte, Jung Suk Lee, Kim D. Thompson, Hyeongsu Kim, Tae Sung Jung

**Affiliations:** 1 Laboratory of Aquatic Animal Diseases, Research Institute of Natural Science, College of Veterinary Medicine, Gyeongsang National University, 501-201, 501, Jinju-daero, Jinju-si 52828, Korea; wjdwodnr0605@gmail.com (J.W.J.); gladofls@naver.com (A.R.L.); afteru70@gmail.com (J.K.); yl0808@nate.com (Y.R.K.); jassylazarte@yahoo.com (J.M.S.L.); leejs058@gmail.com (J.S.L.); 2 Moredun Research Institute, Pentlands Science Park, Bush Loan, Penicuik EH26 0PZ, UK; Kim.Thompson@moredun.ac.uk; 3 Inland Aquaculture Research, National Institute of Fisheries Science, #55, 25gil, Yeomyeong-ro, Jinhae-gu, Changwon-si 51688, Korea; kimk2k@korea.kr; 4 Centre for Marine Bioproducts Development, Flinders University, Bedford Park 5042, Australia

**Keywords:** helper T cells, cytotoxic T cells, monoclonal antibody, lymphocytes, olive flounder

## Abstract

In higher vertebrates, helper and cytotoxic T cells, referred to as CD4 and CD8 T lymphocytes, respectively, are mainly associated with adaptive immunity. The adaptive immune system in teleosts involves T cells equivalent to those found in mammals. We previously generated monoclonal antibodies (mAbs) against olive flounder (*Paralichthys olivaceus*) CD4 T cells, CD4-1 and CD4-2, and used these to describe the olive flounder’s CD4 Tcell response during a viral infection. In the present study, we successfully produced mAbs against CD8 T lymphocytes and their specificities were confirmed using immuno-blotting, immunofluorescence staining, flow cytometry analysis andreverse transcription polymerase chain reaction (RT-PCR). The results showed that these mAbs are specific for CD8 T lymphocytes. We also investigated variations in CD4 and CD8 T cells populations, and analyzed the expression of immune-related genes expressed by these cells in fish infected with nervous necrosis virus or immunized with thymus dependent and independent antigens. We found that both CD4 and CD8 T lymphocyte populations significantly increased in these fish and Th1-related genes were up-regulated compared to the control group. Collectively, these findings suggest that the CD4 and CD8 T lymphocytes in olive flounder are similar to the helper and cytotoxic T cells found in mammals, and Th1 and cytotoxic immune responses are primarily involved in the early adaptive immune response against extracellular antigens.

## 1. Introduction

In higher vertebrates, the T cells thatplayan essential role in the adaptive immune response are divided into two subsets according to their function, namely helper T cells and cytotoxic T cells [1,2,3]. Helper and cytotoxic T lymphocytes are defined by the expression of CD4 and CD8 glycoproteins on their surface, respectively [4]. The CD4 glycoprotein in mammals consist of four immunoglobulin (Ig)-like extracellular domains (D1–D4), a transmembrane domain and a cytoplasmic tail. Mammalian CD8 is a membrane-bound glycoprotein represented by either homodimers consisting of α-chains, or heterodimers consisting of α-chains and β-chains [5,6]. Both CD8α and CD8βmolecules are comprised of an immunoglobulin (Ig)V-like extracellular domain, a transmembrane domain and a cytoplasmic tail. CD4 and CD8 glycoproteins recognize exogenous antigens by interacting with MHC class II through N terminal domains (D1 and D2) and with MHC class I through IgV-like domain, respectively [3,6,7]. To activate the T cells, signal transduction by intracellular recruitment of Lck kinase occurs in the cytoplasmic tail of CD4 and CD8 glycoproteins [8,9,10].

The presence of CD4-like molecules, CD4-1 and CD4-2, have been investigated in several teleost species, including olive flounder (*Paralichthys olivaceus*), Atlantic salmon (*Salmo salar*), rainbow trout (*Oncorhynchus mykiss*), channel catfish (*Ictalurus punctatus*), fugu (*Takifugu rubripes*), carp (*Cyprinus carpio*) and European sea bass (*Dicentrarchus labrax*) [1,3,7,8,11,12,13,14,15]. The CD4-1 molecule possesses similar components to that of mammalian CD4, containing four Ig-like domains, a transmembrane domain and a cytoplasmic tail. The second CD4-like gene, referred to as CD4-2, on the other hand, has two or three Ig-like domains [16]. The cytoplasmic domain of both teleost CD4-1 and CD4-2 molecules possess an important motif that can bind to p56lck tyrosine kinase, and these CD4 molecules appear to interact with Lck in a similar manner to that observed in mammals. It is also speculated that the CD4 molecules in teleostsmight be involved in the activation of helper T cells through cytoplasmic motifs, which are essential for intracellular signaling after antigen presentation [7,16].

CD8 co-receptors have been well-characterized in mammals, such as human, pig and monkey [17,18,19], as well as in teleosts, including Atlantic halibut (*Hippoglossus hippoglossus*), Atlantic salmon, European sea bass, Gilthead sea bream, carp, fugu, rainbow trout [6,9,20,21,22,23,24]. The teleost CD8-like molecule is divided into two distinct genes, CD8α and CD8β, and ismainly expressed on the surface of T cells as a heterodimer consisting of an α-chain and a β-chain [25]. Both teleost CD8α and CD8β subunits, similar to mammalian CD8 molecules, are composed of an Ig superfamily (IgSf) domain, a hinge region, a transmembrane domain and a cytoplasmic tail [10,25]. It seems that the overall organization of CD8 genes has been conserved throughout the evolution of vertebrates, with the major regions represented similarly in both mammals and fish. However, the C-X-C motif, known as p56lck binding site, can only be found in the cytoplasmic tail of CD8α in mammals, whereas in teleost this particular motif is represented as the C-X-H motif in both CD8α and CD8β [9,23,25].

Although the expression of the CD4 and CD8 genes in teleosts appears similar to that in mammals, identification of their function in teleosts has been impeded by the lack of monoclonal antibodies (mAbs) able to detect CD4 and CD8 molecules [1,3]. While antibodies specific to CD4 lymphocytes and CD8 T cells are present in teleost [26], including olive flounder [1,4,27,28], the detailed functional activities related to CD4 and CD8 lymphocytes are still lacking at a cellular level. Our preliminary studies verified that the mAbs we produced against olive flounder CD4 lymphocytes (i.e., CD4-1 and CD4-2) were specific for these cell types, and different proportions of these CD4 T cells were found in the various tissues examined [1,4]. The aim of the present study, was to produce mAbs able to detect CD8α and CD8β lymphocytes in olive flounder, and to examine the distribution of CD8-positive cells in various tissues of the fish.

In order to have a more comprehensive understanding of the immune response of CD4 and CD8 T cells in olive flounder, we examine the in vivo proliferation of CD4- and CD8-positive T cells in response to nervous necrosis virus (NNV), an infectious disease that causes abnormal behavior and visual dysfunction in marine and freshwater fish [29,30,31,32], and foreign antigens keyhole limpet hemocyanin (KLH) and lipopolysaccharide (LPS), which are considered to be T cell-dependent and -independent antigens, respectively, and capable of initiating an immune response [33,34,35,36]. We also examined the function of Th1 and Th2 cytokines produced in response to NNV, KLH and LPS stimulation, including the expression of Th-specific cytokines and transcription factors.

## 2. Materials and Methods

### 2.1. Fish Husbandry

Healthy olive flounders, weighing between 50 and 80 g, were purchased from local fish farm (Samjin fish farm, Namhae, Korea). The fish were acclimated to laboratory conditions beforecommencing any experiment and fed with dry food pellets once a day. Tissues and peripheral blood were collected from fish for gene cloning, immunofluorescence staining, flow cytometry and reverse transcription polymerase chain reaction (RT-PCR). All tissue samples were used immediately after dissection or stored at −80 °C until required. Prior to dissection and injection, fish were anesthetized by immersion in 0.1 g/L of ethyl 3-aminobenzoate methane-sulfonic acid (Sigma-Aldrich, St.Louis, MO, USA). All experiments were conducted in accordance with the guidelines on animal ethics.

### 2.2. Synthesis of CD8α- and CD8β-Peptides

The nucleotide sequences obtained from NCBI database of CD8α and CD8β from olive flounder (Accession No. AB082957.1 andAB643633.1) were translated using Expasy (https://web.expasy.org/translate/). The signal peptide, transmembrane domain and immunoglobulin-like domains were predicted using Signal P 4.1 (http://www.cbs.dtu.dk/services/SignalP-4.1/), TMHMM 2.0 (http://www.cbs.dtu.dk/services/TMHMM-2.0/) and SMART software (http://smart.embl-heidelberg.de/), respectively [37]. The secondary structures and the physico-chemical characteristics of amino acids, including antigenicity, hydrophobicity, charge density and flexibility were analyzed using DNAStar Protean program. Based on the structural analysis, the extracellular regions of CD8α and CD8β were first selected, and criteria, such as high hydrophilicity, good antigenicity and flexibility, were considered for choosing the immunogenic peptide candidates. Finally, three potential candidates for each protein were synthesized with purity ≥ 95% and conjugated with bovine serum albumin (BSA) (Table 1 and Table 2).

### 2.3. Production of mAbs (3H9-CD8α, 1G7-CD8β) Specific to CD8 Lymphocytes from Olive Flounder

Three peptides (100 µg for each peptide) were mixed and these were used to immunize three 6-week-old female BALB/c mice. The antigens were mixed with Freund’s complete adjuvant (FCA) (1:1 *v*/*v*) for the first round of the immunization. Three subsequent rounds of immunization emulsified with Freund’s incomplete adjuvant (FIA) were performed at two week intervals. Two weeks after the last injection, boosting was performed by injecting 10µgof the un-adjuvanted antigens into the tail vein of the mice. Three days after this booster immunization, spleen cells were harvested from the mice and fused with Sp2/o myeloma cells using polyethylene glycol according to standard protocols [38]. The hybridomas were grown in 96-well plates on a feeder layer of mouse blood cells. Enzyme-linked immunosorbent assay (ELISA) and Western blotting were conducted to select positive hybridomas. The clones that showed the best specificity for the olive flounder CD8αand CD8β antigen were mAb 3H9 and 1G7, respectively. The isotyping of the mAb was performed by ELISA using mAb isotyping reagents (Sigma-Aldrich, St.Louis, MO, USA).

### 2.4. Construction of Plasmids

To generate pKIN/CD4-1, pKIN/CD4-2, pKIN/CD8α and pKIN/CD8β, the whole CD4-1, CD4-2, CD8α and CD8β DNA fragments flanked by two Sfi I sites (Table 3) were amplified and inserted into the two Sfi I sites of the pKINGeo vector (produced in our lab). Protein expression of these plasmids was verified through Western blotting. For immunostaining, the whole CD8α and CD8β DNA fragments flanked by two Sfi I sites were generated and inserted into the p514 vector (produced in our lab) to obtain the p514/CD8α, p514/CD8β.

### 2.5. Transfection

The constructed plasmids were purified using DNA Spin miniprep kits (Intron, Sungnam, Korea) and quantified using a NanoDrop spectrophotometer (Thermo Fisher Scientific, Waltham, MA, USA). Human embryonic kidney (HEK) 293Fcells were seeded into 8-well chamber slides or 24-well plates, grown to a 90% confluence, and transfected with the 2 µg of the constructed plasmids using Lipofectamine2000 (Invitrogen, Carlsbad, CA, USA) according to the manufacturer’s instructions. After 4 h, the transfectants were transferred to Dulbecco’s Modified Eagle’s medium (DMEM) (Thermo Fisher Scientific, Waltham, MA, USA) containing 2% fetal bovine serum (FBS). After 48 h, cells were used for Western blotting, immunofluorescence staining and flow cytometry analysis as described below.

### 2.6. Western Blotting

Different amounts of recombinant CD4-1, CD4-2, CD8α, and CD8β protein samples and 1 × 10^7^ cells/mLof cell suspensions from the spleen, head-kidney and peripheral blood (see Section 2.7 for their isolation), resuspended in RIPA buffer (25 mM Tris-HCl pH 7.6, 150 mM NaCl, 1% NP-40, 1% sodium deoxycholate, 0.1% SDS) (Thermo Fisher Scientific, Waltham, MA, USA). were separated on 12% SDS-PAGE under reducing conditions (80 V for 20 min, 120 V for 90 min). Protein bands in gels were transferred to methanol-activated polyvinylidene fluoride (PVDF) membranes (Thermo Fisher Scientific, Waltham, MA, USA) at 50 mA for 90 min. The membranes were blocked with 5% (*w*/*v*) skimmed milk in 1× phosphate buffered saline (PBS) containing 0.1% (*v*/*v*) Tween 20 (Bio-Rad Laboratories, Hercules, CA, USA), and then incubated with respective mAbs (CD8α, 3H9, CD8β,1G7) followed by horseradish peroxidase (HRP)-conjugated goat anti-mouse IgG (Thermo FisherScientific, MA, USA). These membranes were then visualized using the SuperSignal WestPico Chemiluminescent Substrate kit (Thermo Fisher Scientific, Waltham, MA, USA).

### 2.7. Preparation of Leukocytes from Olive Flounder

Blood sampleswere immediately diluted (1:4) in cold Dulbecco’s Modified Eagle’s medium (DMEM) containing heparin. Other organs, including the gills, liver, spleen, head-kidney, trunk-kidney and intestine, were homogenized and filtered separately througha cell strainer (BD Falcon, Bedford, MA, USA), and the leukocytes were isolated using aPercoll (GE Healthcare, Chicago, IL, USA) gradient [28]. All procedures involving cells werecarried out at 4 °C under sterile conditions. Cell concentration and viability were determined using ahemocytometer.

### 2.8. Flow Cytometry

To examine cell population in various tissues and peripheral blood, a total of 1 × 10^7^ leukocytes per sample from the gill, liver, spleen, head-kidney, trunk-kidney, intestine and peripheral blood were isolated.To analyze leukocyte proliferation after antigen stimulation in vivo, leukocytes from the spleen, head-kidney and peripheral blood were separated as described above and centrifuged at 500× *g* for 3 min. Cells were blocked with 0.1% BSA in 1× PBS for 30 min. Leukocytes were then treated with respective mAbs, followed by Fluorescein isothiocyanate (FITC)-conjugated AffiniPuregoat anti-mouse IgG (Jackson ImmunoResearch, West Grove, PA, USA) for 1 h. Cells werewashed with 1× PBS between each step, resuspended in 1× PBS, then all cells labelled with the mAbs were analyzed by a FACSCaliburTM (BD biosciences, Bedford, MA, USA). At least 30,000 events were measured for each sample.

### 2.9. Immunofluorescence Staining

To identify the reactivity of mAbs to mAb-positive cells, CD8α-positive and CD8β-positive HEK 293F cells were fixed onto 8-well chamber slides with 4%paraformaldehyde (Intron, Sungnam, Korea) for 15 min. Cells were blocked with 0.1% BSA in 1× PBS for 30 min, and stained with anti-CD8α mAb (3H9) or anti-CD8β mAb (1G7) for 1 h, followed by FITC-conjugated AffiniPure goat anti-mouse IgG for 1 h. To confirm leukocytes recognized by mAbs, a final concentration of 1×10^5^ cells from the head-kidney were prepared on a slide glass using a cytological centrifuge (Hanil Science Industrial, Gimpo, Korea) at 30× *g* for 5 min, and then fixed with 4% paraformaldehyde for 15 min, blocked with 0.1% BSA in 1× PBS for 30 min, and stained with anti-CD8α mAb (3H9) or anti-CD8β mAb (1G7) for 1 h, followed by FITC-conjugated AffiniPure goat anti-mouse IgG for 1 h. Cells were then stained with DAPI for 10 min at room temperature. Negative controls were only stained with FITC, and three washes with 1× PBS were carried out between each step. The HEK293Fcells and leukocytes recognized by mAbs (CD8α, 3H9, CD8β, 1G7) were examined under a fluorescence microscope, Olympus FV 1000 (Olympus, Seoul, Korea).

### 2.10. RT-PCR with Flow Cytometry Sorted Leukocytes

Leukocytes (1 × 10^6^ cells/mL in 1× PBS) from head-kidney were prepared and stained as described in the flow cytometry section, and sorted using a FACSARIA III cell sorter (BD Biosciences, San Jose, CA, USA). Lymphocytes from the head-kidney were separated based on3H9-positive and -negative cells and 1G7-positive and -negative cells. Total RNA was extracted from 30,000 sorted cells of each population using an easy-BLUE Total RNA Extraction Kit (Intron, Sungnam, Korea) and reverse transcribed into cDNA using a TOPscript cDNA Synthesis Kit with Oligo (dT) primers (Enzynomics, Daejeon, Korea) according to the manufacturer’s instructions. Specific primers, including CD3ε, CD4-1, CD4-2, CD8α, CD8β, TCRα, TCRβ, IgL, IgM and β-actin were used for the RT-PCR and are shown in Table 4. For the RT-PCR, 1 μL of cDNA template and 10 pM of each primer were used together with an AccuPower ProFi Taq PCR premix (Bioneer, Daejeon, Korea). The PCR conditions were as follows: one cycle of 95 °C for 3 min, 34–40 cycles at 95 °C for 20 s, 55–65 °C as the annealing temperature for 20 s, and 72 °C for 50 s. The PCR products were checked on a 1% agarose gel, then were stained with RedSafe nucleic staining solution (Intron, Sungnam, Korea). Images were visualized by an AE-9000E graph(ATTO Corporation, Tokyo, Japan). Each analysis was repeated three times.

### 2.11. Assessing Populations of CD4-1-, CD4-2-, CD8α- and CD8β-Positive Lymphocytes in Olive Flounder

Fish were separated into four groups; NNV infected (1 × 10^7^ TCID50/mL), KLH injected group (100 µg), LPS injected (100 µg) and non-treated control; 100 µL of vius/antigen was administered intoeach group of fish, respectively. Fish were infected with NNV particles by intramuscular injection, or administered KLH and LPS by intraperitoneal injection, while the negative control fish were injected with the same volume of 1× PBS. At 0, 1, 3, 7, 14 and 21 days post-administering (dpa) the virus/antigen, the spleen, head-kidney and peripheral blood were collected from three fish in each tank.After isolating the leukocytes, cells were incubated with either 10F8 (anti-CD4-1 mAb), 3C8 (anti-CD4-2 mAb), 3H9 (anti-CD8α mAb) or 1G7 (anti-CD8β mAb), followed by FITC-conjugated AffiniPure goat anti-mouse IgG. The CD4-1-, CD4-2-, CD8α- and CD8β-positive cell populations were compared to the negative controls stained with only FITC-conjugated AffiniPure goat anti-mouse IgG.

### 2.12. Quantitative Real-Time PCR (RT-qPCR) Analysis

At 0, 1 and 3 dpa, five fish from each group were sampled, and head-kidneys collected. Leukocytes (1 × 10^6^ cells/mL in 1× PBS) from the head-kidney were prepared, and cDNA was synthesized from total RNA. The cDNA was diluted 10-fold with RNAse-free water and subjected to reverse transcriptase qPCR (RT-qPCR) to amplify six immune-related genes genes: IFNγ1, STAT-1, T-bet, Perforin, IL-13 and GATA-3 (Table 5). The specificity of each primer set was verified through their dissociation curve. The △△Ct method was utilized to quantify the fold changes in immune gene transcripts within each sample. The expression levels of the target genes were normalized to that of the β-actin, and were expressed as the fold change relative to the average level in the PBS group, which was given the value 1.

### 2.13. Statistical Analysis

The data are displayed as the mean ± standard deviation (SD). Statistical analysis was performedusing a one-way analysis of variance (ANOVA) with GraphPad Prism v8.0 software.

## 3. Results

### 3.1. Synthesis and Selection of CD8α and CD8β Peptides

**Figure 1:**
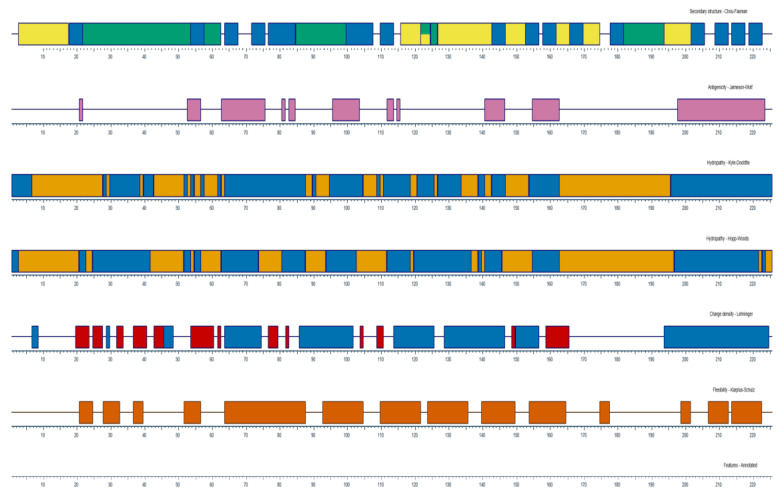
The properties of CD8α molecules analyzed from DNAStar Protean program. The secondary structure, antigenicity, hydrophobicity, charge density and flexibility are represented. Pink, High antigenicity; Blue, Hydrophilic region; Orange, Hydrophobic region.

**Figure 2:**
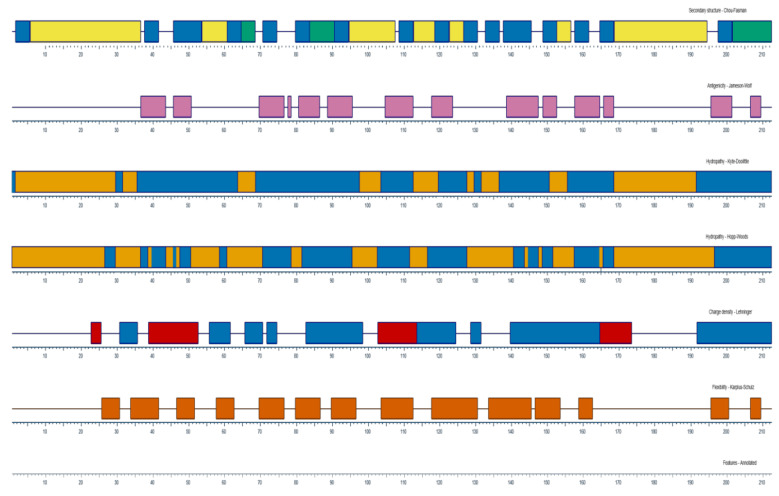
The characteristics of CD8β molecules analyzed from DNAStar Protean program. The secondary structure, antigenicity, hydrophobicity, charge density and flexibility were represented. Pink, High antigenicity; Blue, Hydrophilic region; Orange, Hydrophobic region.

Based on the analysis of CD8α and CD8β amino acid sequences (Figure 1 and Figure 2), three peptides located in the extracellular domain were selected and the amino residues were synthesized as immunogens for immunizing the mice (Table 1 and Table 2). After cell fusion, mAbs showing strong reactivity to the CD8α and CD8β peptide were selected for further analysis; these were mAb3H9 specific for the CD8α peptide and 1G7 specific for the CD8β peptide. The isotype of both mAbs was identified as IgG1.

### 3.2. Reactivity of Anti-CD8α and Anti-CD8β mAbs

**Figure 3:**
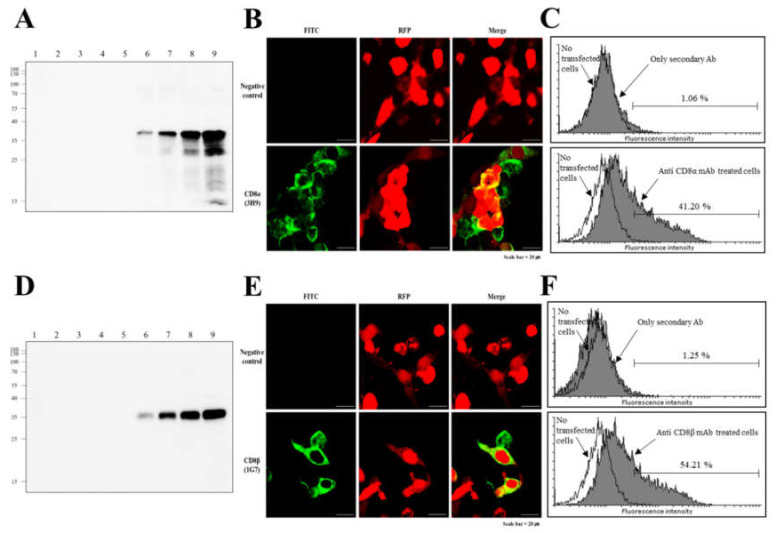
Reactivity of anti-CD8α mAb and anti-CD8βmAb to transfected HEK 293F cells stably expressing flounder CD8αand CD8β molecules. (**A**) The antibody was screened for specificity against increasing amounts of recombinant CD8α. Lanes:(1), rCD4-1 at 500 ng; (2), rCD4-2 at 500 ng; (3), rCD8β at 500 ng; (4), rCD8α at 16 ng; (5), rCD8α at 32 ng; (6), rCD8α at 63 ng; (7), rCD8α at 125 ng; (8), rCD8α at 250 ng; (9), rCD8α at 500 ng. (**B**) Immunofluorescence staining of CD8α-positive HEK 293F cells, with the anti-CD8α mAb (3H9), showing the CD8α molecule located on the surface of the cells, Scale bar = 20 um. (**C**) Compared with the populations of cells expressing the CD8α molecule, a number of cells (~39%) showed reaction to anti-CD8α mAb (3H9), and only ~2% of cells were observed in the negative controls. (**D**) The antibody was screened for sensitivity against increasing amounts of recombinant CD8β. Lanes:(1), rCD4-1 at 500 ng; (2), rCD4-2 at 500 ng; (3), rCD8α at 500 ng; (4), rCD8β at 16 ng; (5), rCD8β at 32 ng; (6), rCD8β at 63 ng; (7), rCD8β at 125 ng; (8), rCD8β at 250 ng; (9), rCD8β at 500 ng.(**E**) Immunofluorescence staining of CD8β-positive HEK 293F cells, with the anti-CD8β mAb (1G7), Scale bar = 20 um. (**F**) Numerous cells (~40%) reacted with anti-CD8β mAb (1G7), and only ~2% of cells were observed in the negative controls.

The specificity of the mAbs were confirmed by Western blot analysis under reducing conditions. The anti-CD8α mAb (3H9) detected different amounts of recombinant CD8α proteins (rCD8α), ranging from 16 ng to 500 ng, corresponding to their estimated molecular weight of ~32 kDa. This mAb did not exhibit any reaction with irrelevant proteins, rCD4-1, rCD4-2 and rCD8β (Figure 3A). Recombinant CD8β proteins (rCD8β), were recognized byanti-CD8β mAb (1G7) at approximately ~31 kDa, at concentrations ranging from 16 ng to 500 ng, whereas other recombinant proteins, including rCD4-1, rCD4-2 and rCD8α were not recognized byvthis mAb (Figure 3D). To determine whether these mAbs (3H9 and 1G7) could detect whole CD8α and CD8β proteins, transfected HEK 293F cells, stably expressing CD8α and CD8β molecules, were incubated with anti-CD8α mAb (3H9) and anti-CD8β mAb (1G7) and examined by confocal imaging and flow cytometric analysis. Staining with the anti-CD8α mAb (3H9) and the anti-CD8β mAb (1G7) was observed at the surface of CD8α- and CD8β-positive HEK 293F cells, respectively, while no fluorescence staining was seen at the surface of negative control cells (Figure 3B,E). When comparing the populations of cells expressing CD8α and CD8βmolecules, ~ 40% of cells reacted with the anti-CD8α mAb (3H9) and the anti-CD8β mAb (1G7), whereas less than2% of cells reacted in the negative control cell population (Figure 3C,F).

### 3.3. Specificity of Anti-CD8α and Anti-CD8β mAbs

**Figure 4:**
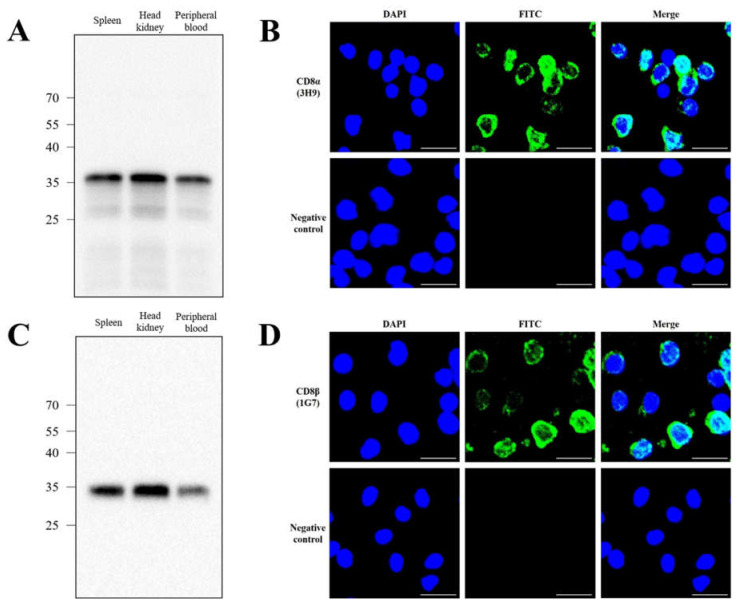
Reactivity of anti-CD8α mAb and anti-CD8βmAb to flounder leukocytes. (**A**) Leukocytes from the spleen, head-kidney and peripheral blood of olive flounder were subjected to Western blot analysis, and the bands recognized by the anti-CD8α mAb (3H9) were detected. (**B**) For immunofluorescence, leukocytes isolated from head-kidney were stained with anti-CD8α mAb (3H9), followed by FITC. (**C**) Leukocytes from the spleen, head-kidney and peripheral blood were recognized by the anti-CD8β mAb (1G7). (**D**) Immunofluorescence staining of head-kidney leukocytes were stained with anti-CD8β mAb (1G7), followed by FITC.

When leukocytes from spleen, head-kidney and peripheral blood of olive flounder were subjected to Western blot analysis, bands were recognized by anti-CD8α mAb (3H9) and anti-CD8β mAb (1G7) at approximately ~35 kDa (Figure 4A,C). Leukocytes from head-kidney were also recognized by the anti-CD8α mAb (3H9) and the anti-CD8β mAb (1G7) under fluorescence microscope. No fluorescent staining was observed in negative control cells, treated with only FITC. The groups of lymphocytes positively stained with mAb 3H9 and 1G7 displayed green fluorescent staining at the surface of the cells (Figure 4B,D). Together, these results indicate that the mAbs developed in the study, specifically detect lymphocytes and the binding sites of the mAbs appear to be on the cell surface.

### 3.4. Distribution of CD8α- and CD8β-Positive Lymphocytes in Tissues by Flow Cytometry

**Figure 5:**
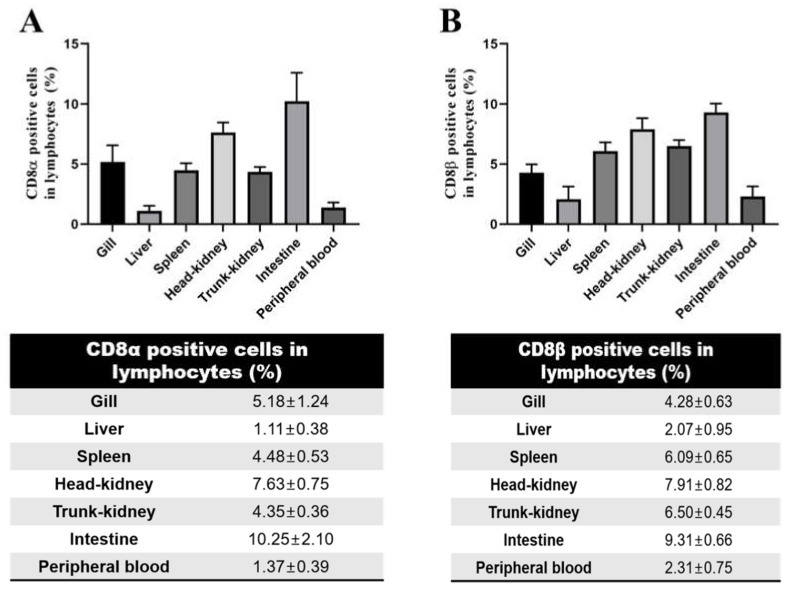
Staining pattern of 3H9 and 1G7 mAbsof leukocytes in different tissues analyzed by flow cytometry. Leukocytes isolated from gill, liver, spleen, head-kidney, trunk-kidney, intestine and peripheral blood were stained with anti-CD8α mAb (3H9) (**A**) or anti-CD8β mAb (1G7) (**B**), followed by FITC. Percentages of CD8α- andCD8β-positive lymphocytes in different tissues, represented as the mean ± SD of at least five fish.

To investigate the proportion of CD8α- and CD8β-positive lymphocytes in various tissues of olive flounder, leukocytes were isolated from gill, liver, spleen, head-kidney, trunk-kidney, intestine and peripheral blood, and analyzed by flow cytometry. Lymphocytes were gated based on the FSC (Forward scatter) and SSC (Side scatter) heights in the dot plot. CD8α-positive cells and CD8β-positive cells in the different tissues were recognized by anti-CD8α and anti-CD8β mAbs (3H9 and 1G7, respectively). Relatively high percentages of CD8α- and CD8β-positive cells were detected in lymphoid tissues, including spleen and kidney, whereas the percentages of CD8α- and CD8β-positive cells in liver and peripheral blood were remarkably low. A relatively high proportion of CD8α and CD8β cells were present in the gill, an important mucosal tissue, and a significantly high proportion was observed in the intestine (Figure 5A,B). The different proportions of CD8α- and CD8β-positive lymphocytes show that distinctive proportions of the two CD8 sub-cells are present in the various tissues examined.

### 3.5. Immune Gene Profiles of CD8α- and CD8β-Positive Lymphocytes

**Figure 6:**
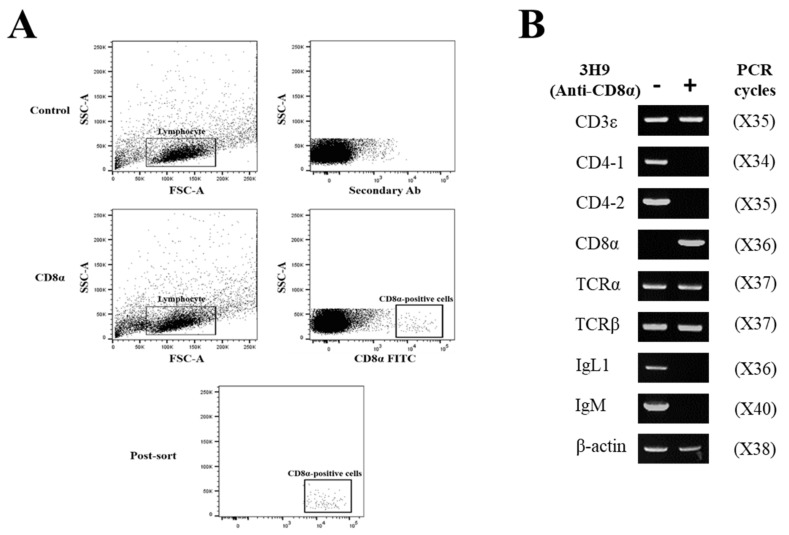
Gene expression profiles of the cell marker genes in flow-sorted spleen and head-kidney lymphocytes. (**A**) Pre- and post-sorting analysis of CD8α-negative and -positive lymphocytes. (**B**) Specific oligonucleotide primers for T cell and B cell gene expression were employed for RT-PCR and β-actin was used as a housekeeping gene. Numbers in the parenthesis indicated PCR cycles.

**Figure 7:**
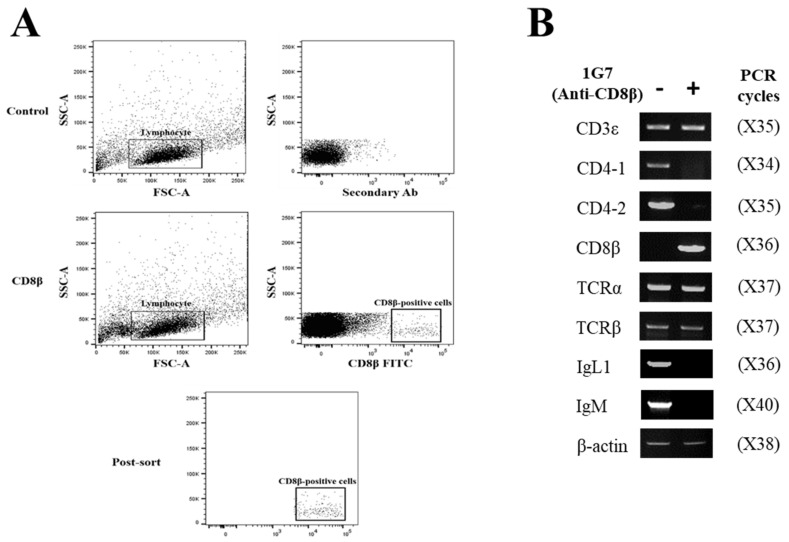
Gene expression profiles of the cell marker genes in flow-sorted spleen and head-kidney lymphocytes. (**A**) Pre- and post-sorting analysis of CD8β-negative and -positive lymphocytes. (**B**) The expression profiles of CD3ε, CD4-1, CD4-2, CD8β, TCRα, TCRβ, IgL and IgM gene transcripts were examined in mAb 1G7-negative and -positive lymphocytes, and β-actin was used as a housekeeping gene. Numbers in the parenthesis indicated PCR cycles.

To examine which cell types reacted with anti-CD8α mAb (3H9) and anti-CD8β mAb (1G7), 3H9-negative and -positive lymphocytes (Figure 6A), and 1G7-negative and -positive lymphocytes in the head-kidney were separated by FACS (Figure 7A), and the expression profiles of CD3ε, CD4-1, CD4-2, CD8α, CD8β, TCRα, TCRβ, IgL and IgM gene transcripts were analyzed. The results revealed that CD8α transcripts were expressed in the 3H9-positive lymphocytes, while they were not detected in the 3H9-negative cells. The CD3ε, TCRα and TCRβ transcripts were amplified in both mAb 3H9-negative and -positive lymphocytes, while the CD4-1, CD4-2, IgL and IgM transcripts were only found in mAb 3H9-negative lymphocytes (Figure 6B). In the case of CD8β lymphocytes, CD8β transcripts were detected in the 1G7-positive lymphocytes, while CD8β transcripts were not found in the 1G7-negative cells. As for the other amplified gene transcripts, the results were similar to that of the CD8α-positive lymphocytes (Figure 7B). Gene transcripts of CD8α- and CD8β-positive lymphocytes were only amplified by T cell-specific primers, not by B cell-specific primers, indicating the specificity of 3H9 and 1G7 was specific to T cells.

### 3.6. Two-Color Flow Cytometry

**Figure 8:**
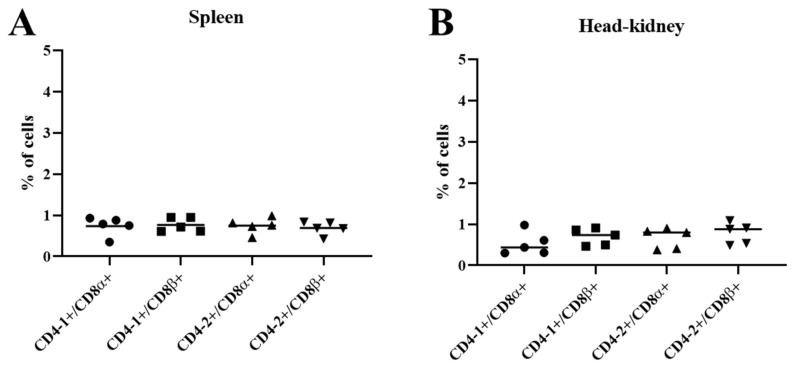
Two color immunofluorescence staining results of CD4^+^/CD8^+^ T lymphocytes in spleen and head-kidney leukocytes. (**A**)Percentage of CD4^+^/CD8^+^cells in spleen. (**B**) Percentage of CD4^+^/CD8^+^cells in head-kidney. Data represented as mean ± SD of positive cells over total lymphocytes of at least five fish.

Expression of CD4/CD8 double positive cells was examined by flow cytometry using anti-CD4 mAbs and anti-CD8 mAbs. Two-color immunofluorescent staining revealed that CD8α- and CD8β-positive lymphocytes expressed very low proportions of both CD4-1 and CD4-2 in spleen (Figure 8A) and head-kidney (Figure 8B). These results denote that the expression of CD4 and CD8 T lymphocytes in olive flounder are indeed defined by distinct T cell subsets.

### 3.7. In Vivo Proliferation of CD4- and CD8-Positive Lymphocytes after Viral Infection with NNV or Administration of KLH or LPS

**Figure 9:**
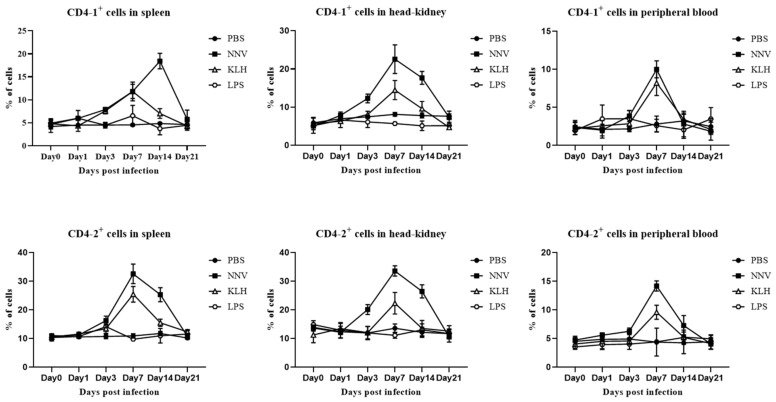
In vivo proliferation of helper T cells after stimulation with NNV, KLH and LPS. Leukocytes from spleen, head-kidney and peripheral blood were collected at 0, 1, 3, 7, 14 and 21 days post-administration, and stained with anti-CD4 mAbs (10F8 or 3C8), followed by FITC-conjugated goat anti-mouse IgG. Lymphocytes were gated on FSC and SSC dot plot and relative cell counts were shown compared to negative control without incubation of anti-CD4 mAbs (10F8 or 3C8). Data are represented as mean ± SD of three analysis.

**Figure 10:**
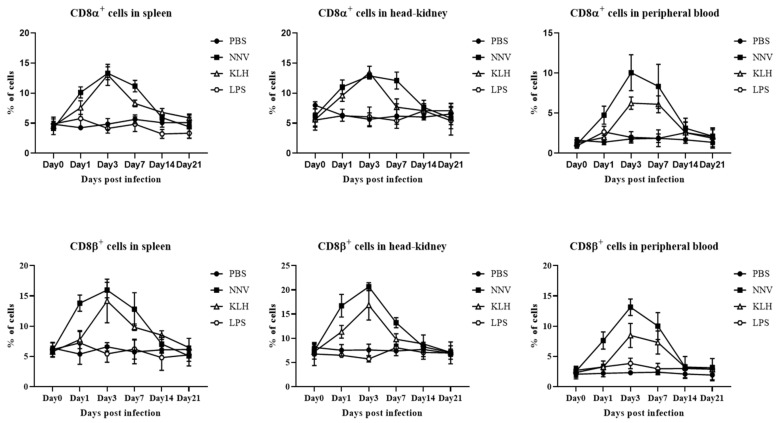
Variations in cytotoxic T cells after injection with NNV, KLH and LPS. Leukocytes from spleen, head-kidney and peripheral blood were obtained at 0, 1, 3, 7, 14 and 21 days post-administration, and incubated with anti-CD8 mAbs (3H9 or 1G7), followed by FITC-conjugated goat anti-mouse IgG. Data are presented as mean ± SD of three analysis.

After administering NNV or KLH, the percentages of both CD4 and CD8 positive lymphocytes showed first an increase, and then after reaching a peak, gradually decreased to base-line levels. The CD4-1 lymphocytes gradually increased and peaked at 14 dpa in spleen and at 7 dpa in head-kidney and peripheral blood, and then started to decrease by 21 dpa. In the case of CD4-2 positive lymphocytes, a significant increase in positively stained cells was observed in spleen, head-kidney and peripheral blood at 7 dpa compared to the control group, and these gradually decreased until the end of the experiment (Figure 9). The proliferation of CD8α- and CD8β-positive lymphocytes appeared from 1 dpa and reached a peak at 3 dpa in spleen, head-kidney and peripheral blood (Figure 10). In the LPS administered group, on the other hand, the population of CD4-1-, CD4-2-, CD8α- and CD8β-positive lymphocytes remained constant over the course of the experiment (Figure 9 and Figure 10). These results imply that T cell-related immune responses could be induced by NNV and KLH, which are T cell stimulants, but not by LPS, which is known to be a B cell stimulant.

### 3.8. Expression of Transcription Factors and Cytokines by Lymphocytes after Virus Infection or Immuno-Stimulation

**Figure 11:**
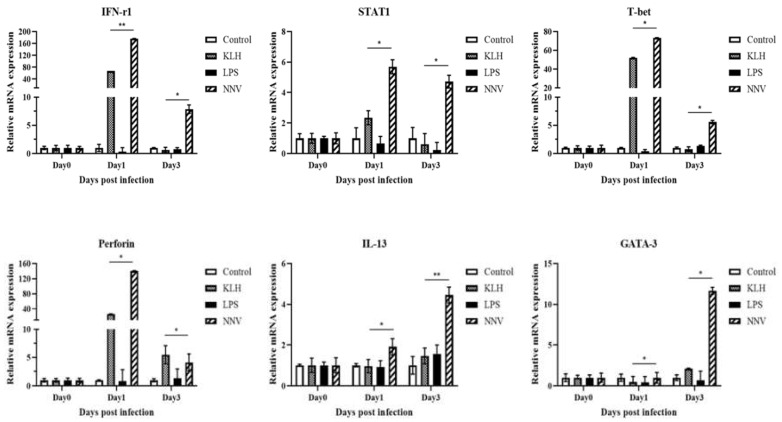
Gene expression analysis of transcription factors and cytokines after stimulation with NNV, KLH and LPS by qPCR. The mRNA level of each gene was normalized to that of β-actin and the error bars represent the standard deviation of results obtained from three fish. The asterisk indicates significant differences compared to the control (* *p* < 0.05, ** *p* < 0.01) based on ANOVA results.

To assess the immune responses associated with helper T cells and cytotoxic T cells after NNV infection, or KLH and LPS immuno-stimulation in olive flounder, the gene expression levels of some transcription factors and several cytokines were analyzed. STAT1, T-bet (both are transcription factors of Th1 cells), and IFN-γ1 (a Th1 cytokine), were increased 1 dpa in the NNV or KLH-treated groups. In particular, the expression level of IFN-γ1 and T-bet showed significant increases from 40- to 180-fold and from 40- to 80-fold, respectively. Along with IFN-γ1, Perforin, one of the effector molecules of cytotoxic T cells, was also increased by approximately 30-fold at 1 day after KLH administration, and more than 120-fold at 1 day post-NNV infection. Subsequently, the expression of GATA-3, the transcription factors of Th2 cells, was up-regulated by 10-fold at 3 days post-NNV infection. IL-13, a Th2 cytokine, also showed a slight increase at 3 days after NNV infection (Figure 11). The results presented here show the dynamics of T cell responses after a viral infection or presentation of thymus dependent or independent antigens in olive flounder.

## 4. Discussion

The gene homologues and structure of helper and cytotoxic T cells in teleosts has now been documented in numerous studies, suggesting that these T cell subsets (i.e., CD4 and CD8 T cells) are similar to those of higher vertebrates [3,39,40]. In mammals, CD4 and CD8 T cells play pivotal roles in the adaptive immune system, especially in the cell-mediated immune response. However, the functional characteristics of teleost CD4 and CD8 T cells are still not well-defined due to the lack of appropriate detection tools like mAbs [1,41]. The function of CD4 T lymphocytes in the immune response against viral infectionswas examined using mAbs developed in previous studies [1,4]. Nonetheless, further studies on the functionality of CD4 and CD8 T cells and comparative studies between these T cell subsets are still required for many fish species. Here, the production and characterization of mAbs against CD8 T lymphocytes were successfully achieved; furthermore, the comparative immune responses between CD4 and CD8 T cells after viral infection and immuno-stimulation were investigated using these newly generated mAbs.

The organization of CD8 genes with the presence of major domains between six exons, is conserved between mammals and teleosts [6,42]. In addition, the entire structure of the cytoplasmic tail has also been preserved between mammals and fish, with only a slight difference. In mammals, the C-X-C motif that interacts with the tyrosine kinase for T cell activation is presented only in CD8α cytoplasmic domain, whereas in teleosts, the corresponding C-X-H motif is observed in both CD8α and CD8β [6,9,23,42,43]. The C-X-H motif with two amino acids, cysteine and histidine in olive flounder can also be observed in other fish species with similar residue placement. Although this teleost motif shows weaker avidity compared to the equivalent motif in mammals, because of the replacement of the second cysteine with histidine, it is speculated that signal transduction occurs in a similar manner with either motif.

The distribution pattern of CD8α- and CD8β-positive T cells observed in the various tissues examined, reflects their tissue localization as immune cells [5]. The CD8 T lymphocyte populations found in lymphoid tissues, including spleen and head-kidney, are relatively high, while consistently low in liver and peripheral blood. These results concur with the role of the spleen and head-kidney as major lymphoid organs of the adaptive immune system in teleosts [44,45]. Interestingly, the percentages of CD8-positive T cells are higher in the gills and intestines by comparison, which are considered as respiratory and digestive organs, respectively. Since fish live in an aquatic environment, various antigens (and pathogens) enter through their gills, and this may explain the prominence of T lymphocytes in the gills of teleost [46,47]. However, in the respiratory organs of humans and mice (i.e., lungs and peri-bronchial lymph nodes), CD8 T cell populations are relatively low [48], thereby, indicating the difference in antigen uptake between teleosts and mammals. Nonetheless, the high distribution of CD8 T cells in the intestine is similar between both of these groups of animals [49,50].

The predominance of CD4/CD8 double positive cells in mammalian thymocyte populations has been demonstrated in previous studies [51,52]. As with mammals, a significant number of CD4/CD8 double positive cells can be detected in the thymus of various fish species, although there are differences in cell populations [5,41]. Nevertheless, the thymus, which is an important lymphoid tissue involved in T-cell development, can only be detected in olive flounder within the first seven months of life, after which thymic involution is observed [45]. Hence, the expression of CD4/CD8 double positive cells were only examined in spleen and head-kidney, where leukocytes are abundant. The results showed the significant low amounts of double positive cells to be present in these organs. This finding agrees with earlier observations that CD4/CD8 double positive cells are absent in lymphoid organs, except for the thymus [41].

The expression profiles of lymphoid cell marker genes were analyzed by RT-PCR using FACS-sorted lymphocytes that reacted with anti-CD8 mAbs (i.e., 3H9 and 1G7). The CD8α-positive lymphocytes expressed transcripts for CD3ε, CD8α, TCRα and TCRβ, while CD8α-negative cells showed the expression of CD3ε, CD4-1, TCRα, TCRβ, CD8α, IgL and IgM transcripts. The expression patterns of the CD8β transcripts were similar to those of CD8α, in which, CD3ε, CD8β, TCRα and TCRβ transcripts were expressed in CD8β-positive lymphocytes, while CD3ε, CD4-1, TCRα, TCRβ,CD8β, IgL and IgM transcripts were detected in CD8β-negative cells. These results, together with those from flow cytometry, indicate that the expression of CD4 and CD8 lymphocyte markers are associated with distinctive T cell subsets in teleosts, as seen in mammals [52,53].

T cells expressing CD4 and CD8 surface proteins, and designated as helper and cytotoxic T lymphocytes, respectively, constitute important components of the adaptive immune system [44]. Although it has been reported that CD4 and CD8 T lymphocytes play crucial roles in virus-induced adaptive immunity, the specific mechanism of helper and cytotoxic T cells during NNV infection is still unclear. Additionally, KLH and LPS are known to stimulate T and B immune cells, respectively, but studies on the immune response related to these molecules are lacking in olive flounder. In the present study, the proliferation of both CD4 and CD8 lymphocytes was observed after NNV infection, and KLH and LPS immune-stimulation. A significant increase in CD4 and CD8 T cells were observed at 7 and 3 days post-NNV infection, respectively. The results concur with the previous observations elaborating that the cell-mediated immune responses occurs within 5 to 7 days after viral infection [54]. Based on our experiment, KLH immuno-stimulation (like NNV infection), can trigger immune responses involving CD4 and CD8 T lymphocytes, specifically because KLH is a T cell-dependent antigen [55]. Furthermore, CD8 T cells proliferated earlier than CD4 T cells in the spleen, head-kidney, and peripheral blood, while CD4-2 cells increased earlier and greater in number than CD4-1 lymphocytes in spleen. This highlights the different roles CD4 and CD8 T cells play in adaptive immunity; moreover, the results also denote that the CD4-2 lymphocytes rather than CD4-1 lymphocytes perform the pivotal role in earlier cell-mediated immune response in olive flounder.LPS immuno-stimulation, on the other hand did not cause proliferation of CD4-1, CD4-2, CD8α, and CD8β T lymphocytes, suggesting that LPS, which is a known B cell stimulant, does not inducean immune response involving T lymphocytes.

The gene expression profiles for dominant cytokines and transcription factors were analyzed in T-cells isolated from head-kidney cells after stimulating the fish with the extracellular antigens. These included T-bet, STAT-1 and GATA-3, three major transcription factors reported in teleost fish, and IFN-γ1 and IL-13, cytokines secreted by Th1 cells and Th2 cells, respectively [7,56,57]. In addition, perforin, an important effector molecule of cytotoxic T lymphocytes (CTLs), NK and NKT cells, along with IFN-γ1, also produced by CD4-positive Th1 cells [5], were also analyzed. In this study, the expression of T-bet and IFN-γ1 showed a significant increase, and subsequently STAT-1 expression was up-regulated at 1 day post-infection with NNVand immuno-stimulation with KLH, whereas slight increases in IL-13 and GATA-3 expression were noted at 3 days post-NNV infection. The fact that up-regulation of the Th1-related immune genes increased more and earlier than the Th2-related genes, suggests that the immune response related to Th1 cells might have a more important role in earlier events than the Th2 immune response during NNV infection and KLH immuno-stimulation. Moreover, these results support previous findings elucidating the reciprocal control mechanisms present between Th1 and Th2 cells [58]. Based on our results, the immune response initiated by CTLs is characterized by the proliferation of helper T cells, as well as increased expression of IFN-γ1 and perforin transcripts. Hence, our data show CTLs to have a fundamental role in the early stage of cell-mediated immune responsesagainst extracellular antigens.

In conclusion, we successfully produced anti-CD8α (3H9) and anti-CD8β (1G7) mAbs that specifically detect CD8 lymphocytes in olive flounder. Using these mAbs, we were able to elucidate that the structure and characteristics of CD8-positive T cells in olive flounder correspond to their mammalian counterparts. The distribution pattern of T lymphocytes in various tissues after introduction of extracellular antigens explains the functional role of helper and cytotoxic T cells, further indicating the direct involvement ofCD4 and CD8 T cells inthe cell-mediated immune response. Particularly, the intensive proliferation of CD8 and CD4-2 T cells and up-regulation of Th1-related genes at the onset of extracellular stimulation signify the synergistic role of CD4-2 and cytotoxic T lymphocytes in the initiation of immune response in olive flounder. These newly produced CD4 and CD8 T lymphocyte-specific mAbs are essential detection tools that can be utilized to expand our knowledge of the functional roles of Th and CTLs in teleosts.

## Figures and Tables

**Table 1:** Synthesized peptides of CD8α molecules in olive flounder.

No	Start	End	Peptide Sequence	Length
1	37	46	CKPAEMFNTV	10
2	60	72	**IASFGRDGKMKSN**	13
3	107	114	TIIQSNEM	8

**Table 2:** Synthesized peptides of CD8β molecules in olive flounder.

No	Start	End	Peptide Sequence	Length
1	44	53	CNCNNSCDSV	10
2	68	80	**LGKCNNAERVNYG**	13
3	116	123	VLKAKSGT	8

**Table 3:** Oligonucleotide sequence of whole DNA fragments for plasmid construction.

Primer Name		Sequence (5′-3′)
CD4-1	Forward	TATAGGCCACCGGGGCCATGGAGAAGTTTGTCCTCATTC
	Reverse	GGTGGGCCCCAGAGGCCTGTTCTGTAGAATCCTTTGGGC
CD4-2	Forward	TATAGGCCACCGGGGCCATGAACGTCATTGTGTTGTTTGGA
	Reverse	GGTGGGCCCCAGAGGCCCTCCTTTAGCAGGGGCTTCAG
CD8α	Forward	TATAGGCCACCGGGGCCATGGACCAAAAGTGGATTCAG
	Reverse	GGTGGGCCCCAGAGGCCAACATGTGTGTTGTTCTTCATCTG
CD8β	Forward	TATAGGCCACCGGGGCCATGAACCCGCTGCCGCTG
	Reverse	GGTGGGCCCCAGAGGCCGGGCATCTGTCTCATCTTCTG

**Table 4:** Oligonucleotide primer sequences used for RT-PCR analysis.

Primer Name		Sequence (5′-3′)
CD3ε (AB081751.1)	Forward	ATGAAAATCAACACCATGGATGTC
	Reverse	TCCCGTCCTGTTCACAATAGA
CD4-1 (AB643634.1)	Forward	ATGAATCCCAGAGGAGAGATAATG
	Reverse	CACGTAGTCTCCTCCGTCTTC
CD4-2 (AB640684.1)	Forward	GTGATCCTAACAAAACCCAGGCAG
	Reverse	AGCAGGTTCTTCAACTTTGATCTT
CD8α (AB082957.1)	Forward	ATGGACCAAAAGTGGATTCAGATG
	Reverse	AACATGTGTGTTGTTCTTCATCTG
CD8β (AB643633.1)	Forward	ATGAACCCGCTGCCGCTG
	Reverse	GGGCATCTGTCTCATCTTCTG
TCRα (AB053227.1)	Forward	ATGCTCTCACTGCATCTTGGT
	Reverse	GACTCTGTGACTGAGCCACAG
TCRβ (AB053228.1)	Forward	ATGATTCCAAGCCTCAACACC
	Reverse	GTGGTTCTGCTTCTCAGCTGA
IgL1 (AB819734.1)	Forward	ATGAGCTTTACCTCCGTCCTC
	Reverse	GGACTGGGAACACTGGTCTCT
IgM (AB052744.1)	Forward	ATGTTTCCTGTAGCTGTGCTG
	Reverse	CTGGGCCTTGCATGGTATGTT
β-actin (HQ386788.1)	Forward	ATGGAAGATGAAATCGCCGCA
	Reverse	GAAGCATTTGCGGTGGACGAT

**Table 5:** The peak values of CD4-1, CD4-2, CD8α and CD8β lymphocytes after nervous necrosis virus (NNV), keyhole limpet hemocyanin (KLH) and lipopolysaccharide (LPS) administration.

		NNV		KLH		LPS	
		DPI (Days post infection)	Peak (Mean ± SD)	DPA (Days post administration)	Peak (Mean±SD)	DPA (Days post administration)	Peak (Mean±SD)
CD4-1 lymphocytes	Spleen	14	18.44 ± 1.36 *	7	11.82 ± 1.66 *	7	6.53 ± 1.84
	Head-kidney	7	22.59 ± 3.06 *	7	14.50 ± 2.04 *	1	6.63 ± 0.56
	Peripheral blood	7	10.00 ± 0.90 *	7	8.21 ± 1.3 4 *	3	3.52 ± 0.87
		NNV		KLH		LPS	
		DPI (Days post infection)	Peak (Mean±SD)	DPA (Days post administration)	Peak (Mean±SD)	DPA (Days post administration)	Peak (Mean±SD)
CD4-2 lymphocytes	Spleen	7	32.59 ± 2.79 *	7	25.48 ± 2.21 *	14	10.99 ± 2.08
	Head-kidney	7	33.62 ± 1.47 *	7	22.36 ± 3.08 *	0	14.89 ± 1.11
	Peripheral blood	7	14.19 ± 0.72 *	7	9.60 ± 1.00 *	14	5.20 ± 0.38
		NNV		KLH		LPS	
		DPI (Days post infection)	Peak (Mean ± SD)	DPA (Days post administration)	Peak (Mean ± SD)	DPA (Days post administration)	Peak (Mean ± SD)
CD8α lymphocytes	Spleen	3	13.31 ± 0.88 *	3	13.03 ± 1.44 *	1	5.77 ± 1.25
	Head-kidney	3	12.87 ± 0.41 *	3	13.41 ± 0.87 *	14	7.07 ± 0.96
	Peripheral blood	3	10.06 ± 1.83 *	3	6.26 ± 0.63 *	1	2.67 ± 0.57
		NNV		KLH		LPS	
		DPI (Days post infection)	Peak (Mean ± SD)	DPA (Days post administration)	Peak (Mean ± SD)	DPA (Days post administration)	Peak (Mean ± SD)
CD8β lymphocytes	Spleen	3	15.98 ± 1.05 *	3	14.17 ± 2.94 *	1	7.24 ± 1.53
	Head-kidney	3	20.66 ± 0.68 *	3	16.83 ± 2.50 *	7	8.05 ± 0.61
	Peripheral blood	3	13.13 ± 1.11 *	3	8.47 ± 1.62 *	1	3.85 ± 0.71

* Represented the statistical significant difference when compared to that in control group (ANOVA, *p* <0.05).

## Data Availability

All data generated or analyzed during this study are included in this published article.
